# MicroRoboScope: A Portable and Integrated Mechatronic Platform for Magnetic and Acoustic Microrobotic Experimentation

**DOI:** 10.1002/adrr.202500195

**Published:** 2026-04-20

**Authors:** Max Sokolich, Yanda Yang, Subrahmanyam Cherukumilli, Fatma Ceren Kirmizitas, Sambeeta Das

**Affiliations:** 1 Department of Mechanical Engineering, University of Delaware, Newark, Delaware, USA; 2 Department of Animal & Food Sciences, University of Delaware, Newark, Delaware, USA

**Keywords:** accessibility, experimentation system, microrobotic actuation, portability

## Abstract

This paper presents MicroRoboScope, a portable, compact, and versatile microrobotic experimentation platform designed for real-time, closed-loop control of both magnetic and acoustic microrobots. The system integrates an embedded computer, microscope, power supplies, and control circuitry into a single, low-cost, and fully integrated apparatus. Custom control software developed in Python and Arduino C++ handles live video acquisition, microrobot tracking, and generation of control signals for electromagnetic coils and acoustic transducers. The platform’s multi-modal actuation, accessibility, and portability make it suitable not only for specialized research laboratories but also for educational and outreach settings. By lowering the barrier to entry for microrobotic experimentation, this system enables new opportunities for research, education, and translational applications in biomedicine, tissue engineering, and robotics.

## 1 | Introduction

Microscale robots have a variety of potential applications in medicine, environmental monitoring, and tissue engineering, due to their small size and capabilities of sensing and manipulation at the small scale [1]. Recent research has demonstrated their potential in applications ranging from ocular drug delivery and in vitro fertilization to root canal prevention and tumor treatment [2, 3]. The most common actuation methods for microscale robots are acoustic and electromagnetic actuation [4]. Acoustic microrobots, for instance, can be manipulated using sound waves to achieve precise movements, while electromagnetic microrobots rely on magnetic fields for their actuation and control.

Recent advances in magnetically propelled microrobots have demonstrated increasingly sophisticated control and locomotion capabilities. In [5], Zhang et al. created a wearable microrobotic navigation device capable of controlling a microrobot with hand gestures. In [6], Li et al. introduce a claw-like microrobot similar to a red blood cell, enabling better navigation and tracking in blood vessels. Swinging flexible nanomotors are demonstrated in [7], and their gait is studied in an oscillating magnetic field. These developments further motivate the need for accessible experimental platforms capable of rapid experimentation and validation.

Additionally, due to a lack of real-time tracking feedback, traditional open-loop control systems for acoustic and magnetic microrobots often fail to provide the necessary accuracy and reliability required for the above applications [8]. Therefore, it is necessary to integrate closed-loop control, particularly real-time movement feedback, with these actuation systems to significantly enhance their accuracy and performance. Naik et al. have demonstrated control using an acoustic phase array for manipulating micro-objects in air [9]; however, they don’t use magnetic fields, and the approach operates in an open-loop system rather than a closed-loop system. Additionally, in 2019, Youssefi et al. developed a magneto-acoustic system for closed-loop manipulation of objects in air; however, the apparatus is not portable and constrained to a desktop computer [10]. Additionally, the system handles millimeter-scale robots instead of microrobots.

**FIGURE 1 |:**
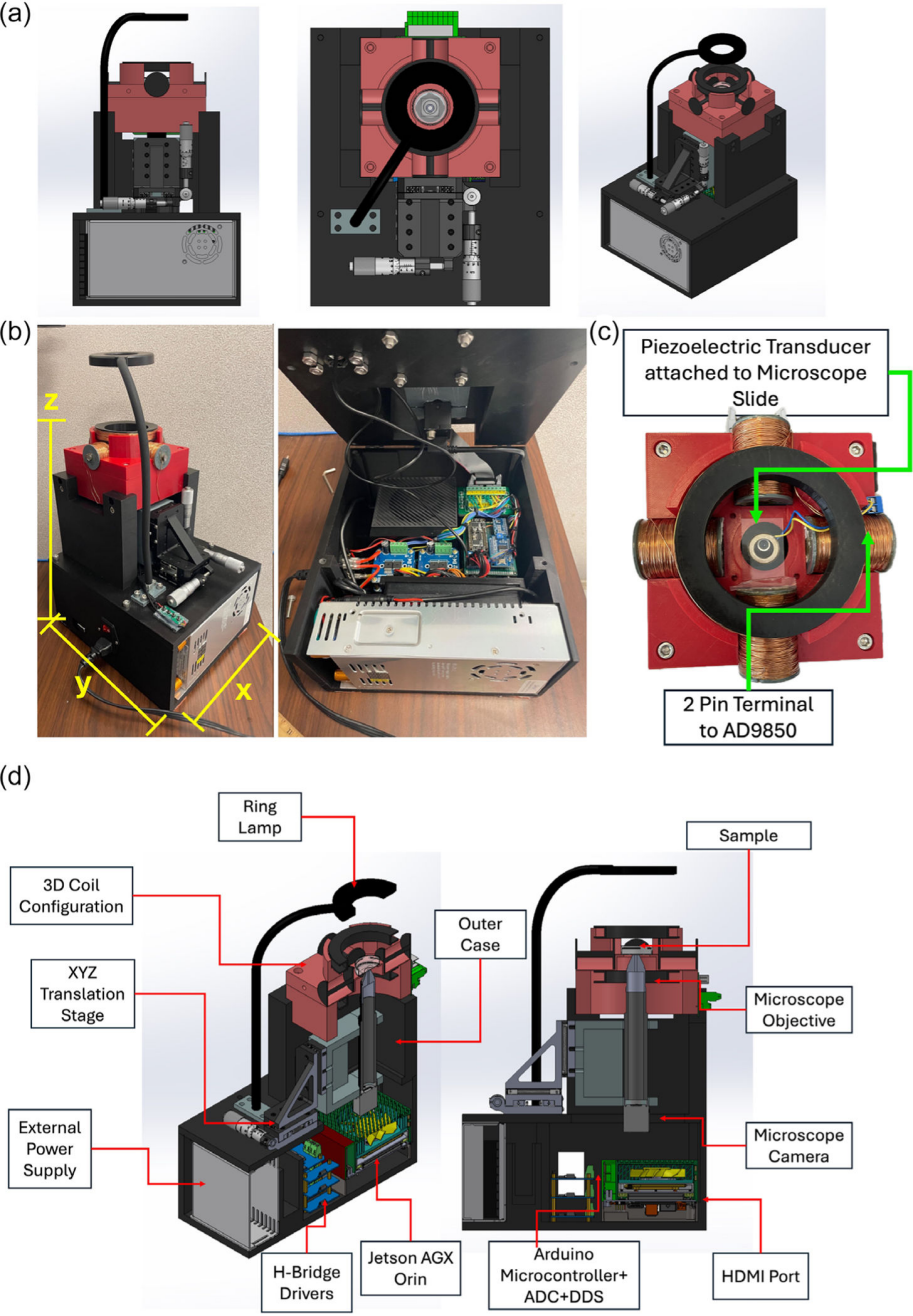
(a) Back, side, front, top, and isometric views of the computer-aided design of the device. (b) Images of the physical system. The system measures 240 mm in the x-direction, 280 mm in the y-direction, and 350 mm in the z-direction. These dimensions allow for all custom-made components, including the outer case, to be 3D printed on an Ender Max 3D printer. All electronics are housed inside the outer case and accessible. (c) Coil configuration with piezoelectric transducer and connections labeled. (d) Computer-aided design of the cross-section of the device with important system components highlighted and defined.

As researchers seek to bridge the gap between laboratory prototypes and real-world applications, compact and portable experimental systems are needed to quickly test the efficacy of microrobots. We have previously developed the ModMag [11], which is a low-cost handheld magnetic microrobot manipulation device. Table 1 summarizes key hardware and integration features of MicroRoboScope in comparison with ModMag. However, this system has no image feedback feature and applies magnetic and acoustic signals entirely in open-loop. Other systems like MicrostressBots have also been developed [12]. However, these systems often require specialized equipment and are not easily transportable, limiting their use in field studies. This paper introduces the development of a compact, portable closed-loop microrobotic experimentation platform, which can combine acoustic and electromagnetic actuation (see Figure 1). The proposed platform integrates real-time visual feedback mechanisms to continuously monitor and adjust the position and velocity of microrobots. The novelty of the system arises from its significant integration of fundamental microrobotic functionality in a compact and portable manner. By leveraging acoustic and electromagnetic control techniques, this platform aims to overcome the limitations of traditional open-loop systems and provide a robust solution for various microrobotic applications. Design choices were guided by the trade-off between compactness, actuation flexibility, and experimental accessibility rather than performance optimization for a single microrobotic task. In summary, the key advantages of the proposed MicroRoboScope platform are: (i) a compact and portable form factor that enables rapid deployment and experimentation across laboratory environments; (ii) integrated 3D electromagnetic and acoustic actuation within a single system; (iii) real-time visual feedback for closed-loop microrobot tracking and control; and (iv) an open-source low-cost unified hardware–software architecture designed to support accessible and reproducible microrobotic experimentation.

## 2 | Background

### 2.1 | Magnetic Actuation

Microrobots are typically actuated via external magnetic fields. By applying an external magnetic field to a magnetic microrobot, the microrobot’s magnetic moment **m** will align with the direction of the applied field. The subsequent torque, **Γ**, is given by

(1) Γ=m×B

where **m** is the magnetic moment of the microrobot and **B** is the magnetic flux density [13]. This torque therefore, allows one to magnetically orient the microrobot in 3D space.

Magnetic forces can also be applied to microrobots by applying magnetic gradients. The magnetic force on a microrobot, **F**, is given by

(2) F=(m⋅∇)B

where **m** is the magnetic moment of the microrobot and **B** is the magnetic flux density [13]. The magnetic forces need to be sufficiently strong to overcome the viscous forces that dominate behavior at the microscale. In practice, it is often difficult to produce adequately strong magnetic forces on microrobots unless the workspace is very small. As a result, it is often advantageous to utilize rotating magnetic fields. These fields result in rolling, spinning, or swimming motion of magnetic microrobots rather than pulling, dragging, or translational motion.

### 2.2 | Acoustic Actuation

Microrobots can also be actuated using external acoustic fields [14]. An acoustic bubble-propelled microrobot typically contains a cavity or hole in its design. When placed in a fluid environment, a gas bubble is trapped in the cavity of the microrobot. The gas bubble is excited via a piezoelectric transducer, which generates bubble propulsion. The microrobot is subjected to various forces under acoustic fields such as acoustic radiation and streaming forces [15]. The acoustic radiation forces include the Primary Bjerknes and Secondary Bjerknes forces [16]. The Primary Bjerknes forces are due to the scattering of the acoustic waves by the oscillating bubble. These are in the same direction as the direction of wave propagation. These forces are negligible due to the attenuation of the waves being much larger than the microrobot size. The two main forces that significantly impact the microrobot motion are the streaming propulsive force and the secondary Bjerknes force [15–17]. The Secondary Bjerknes force creates an attraction of the microrobot to the underlying solid surface. The presence of the boundary results in a boundary condition that can be satisfied by introducing an image bubble (a mirror representation of the real bubble due to the boundary). This image bubble oscillates in phase with the real bubble, producing an attractive interaction force between them [16]. The acoustic streaming forces are generated by the pulsating bubble in the cavity, which creates fluid flows that propel the microrobot away from the open end. These forces are maximized at the resonant frequency of the bubble.

The resonant frequency at which the microrobot moves with its highest velocity is theoretically described via (3) and (4), as follows:

(3) $$f_{0}=\frac{1}{2\pi }{\left(\frac{\kappa P_{0}}{\rho \left(L-L_{b}\right)L_{b}}\right)}^{1/2}\times M$$

(4) $$M={\left(1+\frac{4\gamma L_{b}}{\kappa P_{0}a^{2}}\right)}^{1/2}$$

where κ~1.4 is the adiabatic index, ρ is the density of water, $P_{0}$ is the pressure in the bubble without an acoustic field, $L_{b}$ is the length of the bubble, L is the cavity length, a is the inner radius of the cavity, and γ~0.07N/m is the surface tension of the water–air interface [16].

By tuning the transducer frequency, we adjust how closely the bubble is driven to its resonant state, allowing us to control the microrobot’s speed. This provides a flexible means of acoustic microrobotic actuation.

## 3 | Results

First, we outline the mechanical, electrical, and software specifications of the system. Then, we describe the method of magnetic field generation. Next, we showcase measurements of the magnetic field generated by the system. Subsequently, three microrobotic actuation experiments were conducted to demonstrate the versatility of the system for microrobotic experimentation. We demonstrated the system’s ability to generate a rotating field that results in the autonomous translational rolling motion of a magnetic microrobot alonga circular path. Wealso demonstrate the ability tocontrol the rotating field and, therefore, the rolling microrobot manually with a joystick controller. We show precise manipulation by pushing nonmagnetic passive microspheres while simultaneously tracking the position and velocity. We also demonstrate the system’s ability to generate both magnetic fields and acoustic fields simultaneously within a closed-loop. In this experiment, a 3 μm cup-shaped acoustic microrobot is autonomously guided to a target coordinate in the workspace by regulating the acoustic frequency, microrobot’s velocity, and magnetic field orientation direction in real-time. Finally, we end with a conclusion.

### 3.1 | Mechanical System Specifications

The system measures 240 mm in the x-direction, 280 mm in the y-direction, and 350 mm in the z-direction. The 3D coil configuration consists of 4 standard solenoids arranged about the XY plane, and a pair of Helmholtz coils in the z-direction. This coil configuration allows for 3D magnetic fields to be generated in the workspace. The specifications of these coils are as follows. The core of the standard solenoids is made of electrical steel with a diameter of 8 mm and a length of 45 mm. The relative magnetic permeability of the core is approximately 4000. The outer diameter of the entire coil measures 35 mm, resulting in 1500 turns of 24 AWG copper wire. Opposite-facing solenoids have a separation distance of 50 mm, allowing a standard 35 mm petri dish to sit comfortably at the center, or half of a standard 75 mm by 25 mm glass microscope slide. The pair of Helmholtz coils has an inner diameter of 70 mm and an outer diameter of 100 mm. The thickness of the coil is 10 mm, and there are 400 turns of 24 AWG copper wire. The two coils are placed on the top and bottom of the 4 standard coils, resulting in a coil separation distance of 35 mm. All 6 coils are mounted on a custom stand, designed in Solidworks and printed using an Ender 3 Max 3D printer. The 3D coil configuration is then mounted onto the outer case of the device using standard M6 bolts.

The microscope aspect of the device includes a LX30/M-Self-Contained XYZ 25 mm Translation Stage mounted to a FLIR BFS-U3–50S5C-C USB 3.1 Blackfly S Color Camera. This allows for precise focusing of the substrate using the *Z*-axis micrometer, and translational scanning of the workspace using the *X*- and *Y*-axis micrometers. To mount the camera to the stage, a custom adapter is designed and 3D printed. Then, C-mount extension tubes are used to establish an adequate optical tube length between the camera sensor and a 10x objective lens. The XYZ translation stage is mounted to the outer case below the 3D coil configuration.

The entire outer case is printed in halves and attached with hinges, allowing for convenient inspection of the electric components when necessary (see Figure 1b). Finally, an adjustable ring lamp is attached to the outer case to illuminate the sample. The ring lamp’s brightness can be adjusted, along with options for white, warm-white, and warm-yellow light output.

### 3.2 | Electrical System Specifications

**FIGURE 2 |:**
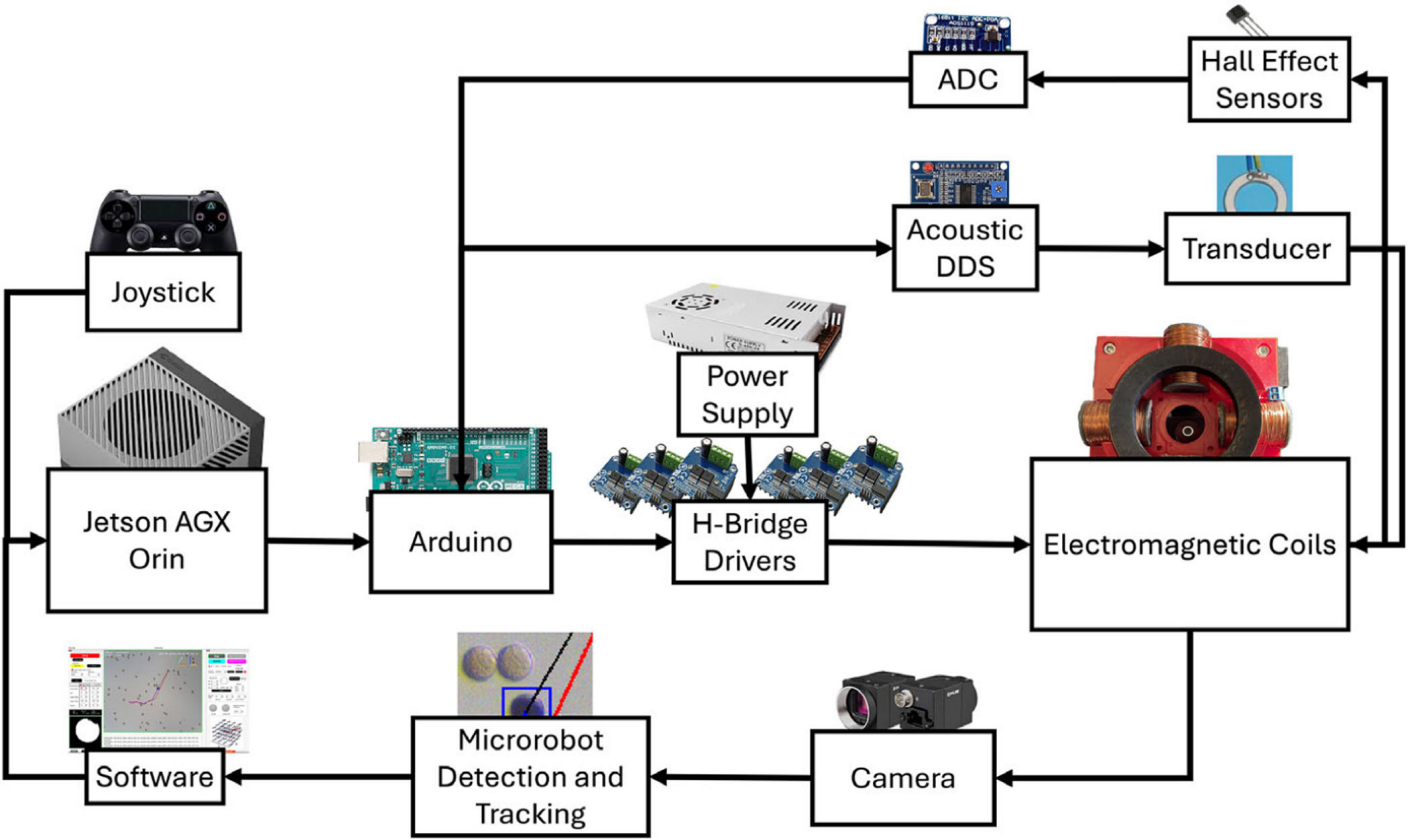
Flowchart of electrical system components. The Jetson AGX Orin communicates with an Arduino over Serial to generate appropriate PWM (Pulse Width Modulation) signals. H-bridge circuits connected to an external power supply are used to switch the low-current PWM signals from the Arduino into high-current signals needed to power the coils. The Arduino also interfaces with an ADS1115 analog-to-digital converter to read Hall effect sensor data, as well as an AD9850 signal generator module to output the high-frequency sine waves needed to drive a piezoelectric transducer. The image feed from a microscope camera captures the microenvironment workspace, enabling tracking and detection of microrobots.

The outer case houses the electrical components required for device operation. To begin, a Nvidia Jetson AGX Orin acts as the host computer for the system. The Jetson Orin single board computer enables the use of powerful parallel computing and GPU functionality, which can enable more advanced microrobotic machine learning applications in future work. A FLIR BFS-U3–50S5C-C USB 3.1 Blackfly S Color Camera is connected to the computer via USB. In order to separate the output control signal thread from the camera processing thread, an Arduino Mega microcontroller is connected to the Jetson Orin via USB. The Arduino is connected to six BTS7960B 43A motor H-Bridge PWM drivers, one for each of the 6 coils. These are used to modulate the large currents necessary to power the electromagnetic coils, as well as switch the polarity of the field. An external 0–27 V, 0–20 A adjustable power supply is used to supply the necessary power to the coils. The external power supply is connected to the H-Bridge drivers. To output the high-frequency sine waves necessary to actuate a piezoelectric transducer and thus an acoustic microrobot, a HiLetgo DDS AD9850 Signal Generator Module is connected to the Arduino. This module is capable of generating sinusoidal waveforms from 0 to 40 MHz at approximately 1 VPP and square waves from 0 to 1 MHz at approximately 1 VPP. These frequency ranges are more than adequate for experimenting with a wide variety of acoustic microrobots. Furthermore, an ADS1115 16-Bit 16-Byte 4-Channel I2C IIC Analog-to-Digital ADC is connected to the Arduino. The ADC is connected to 3 Hall effect sensors attached to the *x*, *y*, and *z*-axis coils for measuring the respective magnetic fields in real-time. Additionally, a PS4 DualShock wireless gaming controller is connected to the Jetson Orin over Bluetooth and used to manually control the generated fields and, therefore, the microrobots in the workspace. Finally, the Jetson can be connected to a monitor via HDMI to run the software. See Figure 2 for the electrical and system component pipeline of the system. See the Supporting Information for a more detailed pin-out representation of the wiring diagram.

### 3.3 | Software System Specifications

**FIGURE 3 |:**
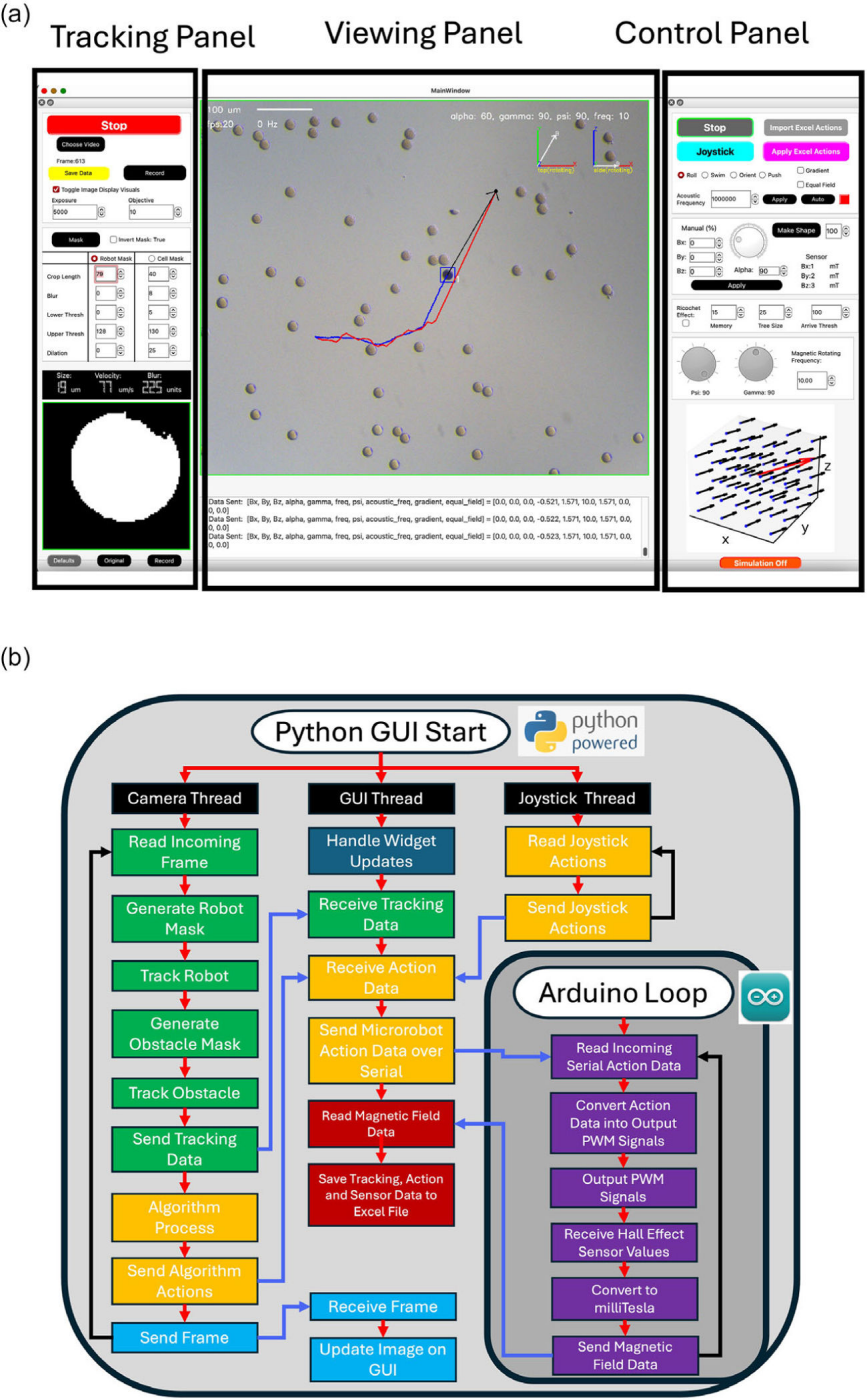
(a) Front-end software architecture developed using the PyQt5 library in Python, with tracking, viewing, and control tabs highlighted. (b) High-level back-end software architecture flowchart illustrating the main Python GUI processes and corresponding Arduino control loop functionality. Arduino and Python logos included to indicate software/hardware used. Logos are trademarks of their respective owners.

A user-friendly control application graphical user interface was designed in order to easily interact with the camera and adjust control signals. The software is written in Python and is initially built using the QT Designer toolbox from the PyQt5 library, which enables simple drag and drop placement of common GUI widgets like buttons and input boxes [18]. The software front-end architect-ture can be split into a tracking panel (left), viewing panel (middle), and control panel (right) (see Figure 3a). Additionally, Figure 3b outlines the high-level, back-end architecture where black arrows indicate loops within a thread, blue arrows indicate data transmission between threads, and red arrows represent the sequential execution of key functions within individual threads. This separation enables real-time perception, logging, and actuation updates while maintaining a responsive user interface.

The tracking panel (left) provides the perception and state-estimation layer for closed-loop microrobot control. Incoming color frames are processed through a lightweight segmentation pipeline consisting of region-of-interest (ROI) cropping, grayscale conversion, thresholding, and optional morphological operations (e.g., blur/dilation) to generate a binary mask. The microrobot pose is estimated using centroid localization on the masked ROI, and the ROI is re-centered online to maintain robust tracking under motion. This centroid-based tracker offers a transparent and computationally efficient baseline that can be tuned to accommodate diverse microrobot appearances and background conditions. Microrobot velocity is estimated via finite differences over a 15-frame temporal window, chosen to balance measurement noise and temporal resolution. The panel also supports camera exposure adjustment and microscope magnification entry to maintain a consistent pixel-to-metric conversion for quantitative analysis. Tracking outputs (e.g., mask parameters, position, velocity, and apparent size per frame) are time-stamped and can be exported for post-processing.

The control panel (right) implements the actuation and control layer, exposing both manual and automated interfaces. For magnetic actuation, the software provides parameterized field primitives including uniform orientation fields, rotating fields for swimming and rolling behaviors, and gradient fields. These primitives are specified by direction, magnitude, and (when applicable) rotation frequency. For acoustic actuation, the panel configures and applies continuous-wave excitation via the AD9850 signal generator module that drives the piezoelectric transducer. Frequency commands can be issued interactively or executed as scripted sequences. An optional online frequency sweep utility is provided to rapidly identify resonant actuation bands for bubble-propelled microrobots. To support reproducible experiments, actuation commands are synchronized with the tracking stream and logged alongside the estimated microrobot state. The panel also offers teleoperation via a game controller, playback of predefined action sequences from external files, and a modular entry point for closed-loop controllers that consume real-time state estimates and generate actuation commands. A compact visualization of the commanded magnetic field vector is rendered to provide immediate feedback on actuation direction and mode.

The viewing panel (middle) streams the microscope image (2048 × 2448 pixels at 24 fps) and overlays key telemetry to support real-time interpretation and debugging. Displayed information includes the camera frame rate, scale bar, applied acoustic frequency, and the commanded magnetic field mode and parameters (e.g., field direction components and rotation frequency). Interactive tools in the viewing panel enable selection of a microrobot for tracking, definition of waypoints/paths for closed-loop navigation, and zooming for inspection of fine structures. A lightweight log console below the image reports the currently commanded actuation signals and system status messages, facilitating reproducibility and troubleshooting during experiments.

The software is cross-platform (Linux, macOS, and Windows) and can run on standard desktop/laptop computers in addition to the Jetson AGX Orin computer host. Deployment requires only USB connectivity to the camera and the microcontroller, which simplifies replication across laboratories and supports portable use cases.

### 3.4 | Magnetic Field Generation

**FIGURE 4 |:**
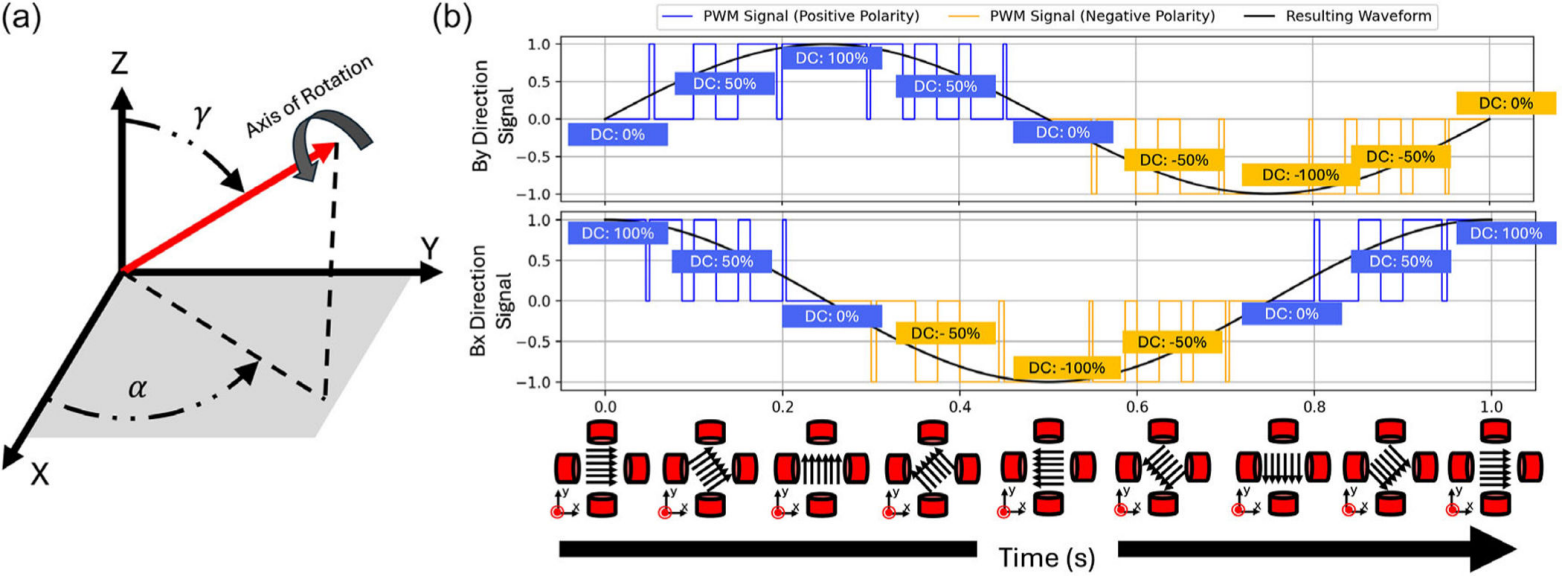
(a) Illustration of the spherical coordinate system used to define the axis of rotation of the rotating magnetic field, as described by Equations (1)–(3). The axis orientation is controlled using the angles α and parameters. Typically $\gamma =90^{\circ }$ for 2D rolling motion. (b) Schematic illustrating how a rotating magnetic field is synthesized using pulse-width modulation (PWM). The two plots show the resulting waveform formed by modulating the DC of PWM signals over time. For the positive portion of the waveform (black sinusoid), the PWM signal is applied while the H-bridge is set to positive polarity (shown in blue). For the negative portion, the PWM is applied with the H-bridge in negative polarity (orange). The PWM signals effectively approximate the target sinusoidal waveform by varying the DC from 0% to ± 100%. In this example, two sinusoidal waveforms are generated: the top signal is applied to the *y*-axis coils and the bottom to the *x*-axis coils, while the *z*-axis field is set to zero. This configuration creates a 1 Hz rotating magnetic field in the XY plane, as illustrated at the bottom. This is equivalent to setting $\gamma =0^{\circ }$ using Equations (1)–(3).

The magnetic field action variables described above are sent to the Arduino using the PySerialTransfer Python library. The Arduino then converts these variables into magnetic field vectors. Then these vectors are applied to the system using PWM duty cycle (DC) modulation [19]. By varying the DC of the PWM signal, we can control the intensity of the magnetic flux density. To generate a uniform field in the x-direction, for example, we actuate the right and left configured coils simultaneously. This generates a pseudo uniform field at the center of the workspace. To generate a gradient magnetic field, we only actuate one coil at a time. For example, to generate a gradient field in the positive x-direction, we actuate the right coil. Equations (5), (6), and (7) define three software-generated sinusoidal waves applied to each axis of the coil system, which generate a rotating magnetic field.

(5) $$B_{x}=-\cos(\gamma )\cos(\alpha )\cos(2\pi ft)+\sin(\alpha )\sin(2\pi ft)$$

(6) $$B_{y}=-\cos(\gamma )\sin(\alpha )\cos(2\pi ft)-\cos(\alpha )\sin(2\pi ft)$$

(7) $$B_{z}=\sin(\gamma )\cos(2\pi ft)$$

where $0<\alpha <360^{\circ }$, $0<\gamma <180^{\circ }$, and 0<f<250 Hz. See Figure 4a for a schematic representation of how these variables adjust the axis of the rotating field. The azimuthal angle (α), polar angle (γ), and frequency (f) of the rotating field can be adjusted in the software.

These sinusoidal waveforms are generated artificially through PWM DC modulation in the Arduino “void loop” function and sampled through time by letting t=micros()/1e6. This is done by calculating the value of $B_{x}$, $B_{y}$, and $B_{z}$ at each loop of the Arduino program. The amplitude of the sine waves is normalized between −1 and 1. These values are then applied using Arduino’s AnalogWrite() function to modulate the PWM DC applied to each H-Bridge driver, which adjusts the field strength in time. Figure 4b conceptually illustrates the waveform generation of a 1 Hz rotating magnetic field in the XY plane. This waveform would spin a magnetic microrobot at 1 Hz in the counterclockwise direction. The blue and orange signals illustrate how varying the DC of the PWM signal can construct an arbitrary waveform. When the PWM signal is blue, we are generating a magnetic field in the positive direction. When the PWM signal turns orange, we are generating a magnetic field in the negative direction. This is done by switching the polarity of the current applied to the H-Bridge drivers when the amplitude of the wave-forms changes sign. Refer to the Arduino code provided in the Supplemental Information for more details. Additionally, Figure 4b also illustrates how a 1 Hz rotating magnetic field signal can be used to spin a magnetic microrobot.

We found that the Arduino program loops at a rate of 500 times each second. This, along with the PWM frequency rate, sets an upper limit to the maximum practical sinusoidal frequency that can be applied. The Arduino Mega PWM frequency was increased from the default 490 Hz to 31 kHz to maximize the potential artificial sine wave resolution that could be sampled. At a rotating magnetic field frequency f=167 Hz, the field output will update every 1/3 of the sinusoidal period. Essentially, the number of data points that make up the sinusoidal signal is given by 500/f. For example, at f=1 Hz, the sinusoidal field will consist of 500 data points. At f=10 Hz, the sinusoidal field will consist of 50 data points. The minimum number of discrete points within one period that can produce a rolling motion of a ferromagnetic microrobot is just over 2, since at 2 or fewer points, aliasing will occur and the field vectors will start to move backward. Therefore, the maximum usable rolling frequency that the system can produce (the Nyquist rate) is approximately 250 Hz, although lower frequencies may be desired to maintain a truer sinusoidal shape of the magnetic field and therefore smoother rotational motion of the microrobot.

### 3.5 | Magnetic Field Characterization

The magnetic field at the center of the workspace for the *x*, *y*, and *z*-axis coils was measured using a TD8620 handheld teslameter. This Tesla meter was used to calibrate each Hall effect sensor as well. The built-in power supply enables the manual adjustment of the supply voltage from 0 V to 27 V. Therefore, the intensity of the magnetic field strength and amplitude of the rotating magnetic field can also be increased or decreased. Additionally, three-dimensional simulations (COMSOL Multiphysics) to evaluate magnetic field uniformity, gradients, and spatial field distributions within the workspace are also simulated. Cross-sectional field profiles for each coil axis are included in the Supporting Information, demonstrating controlled and symmetric field behavior beyond single-point measurements.

**FIGURE 5 |:**
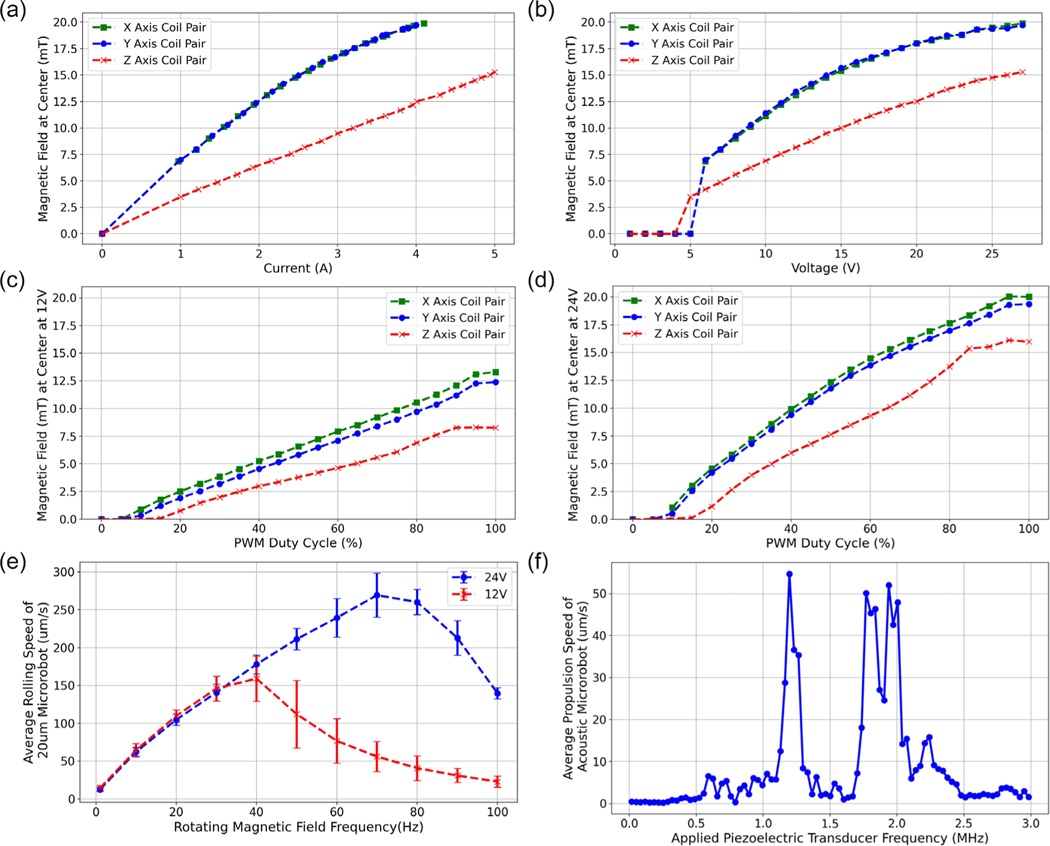
(a) Magnetic field (mT) for the x, y, and z coil pairs as a function of current (A). (b) Magnetic field (mT) for the x, y, and z coil pairs at set power supply voltages from 0 to 27 V at 100% DC. (c) Magnetic field (mT) for the x, y, and z coil pairs at PWM DCs from 0 to 100% at 12V supply voltage. (d) Magnetic field (mT) for the x, y, and z coil pairs at PWM DCs from 0 to 100% at 24 V supply voltage. (e) Average velocity in *μ*m/s of a 20 *μ*m rolling microrobot coated in 250 nm of nickel at 12 and 24 V. (f) Average velocity of a 3 μm cup-shaped microrobot at acoustic frequencies ranging from 0 Hz to3 MHz.

Figure 5b–d shows the results of the measurement experiments. It was found that the magnetic field generated by the *x*-axis coils was comparable to the magnetic field of the *y*-axis coils. Interestingly, the activation voltage of the H-bridge drivers needed to draw a current load from the system’s external power supply was 6 V for the *x* and *y*-axis coils and 5 V for the *z*-axis Helmholtz coil. At the lowest voltage settings, the *x*-axis coils were able to draw 1.0 A, generating a field of 6.9 mT, the *y*-axis coils were able to draw 1.0 A, generating a field of 7.0 mT, and the z-axis coils were able to draw 1.0 A, generating a field of 3.5 mT. Similarly, as we increase the set voltage of the external power supply in 1 V increments, we see a relatively linear increase in the magnetic field at the center of the workspace 5b. However, the linearity for the *x*-axis and *y*-axis coils decreases after approximately 13 V, likely due to heating of the coils, and therefore causes an increased resistance.

The maximum field each axis coil can generate at 27 V at the center of the workspace is 19.9 mT in the x-direction, 19.7 mT in the y-direction, and 15.3 mT in the z-direction. It was found that the magnetic field generated by the pair of Z coils was more linear in shape than the magnetic field generated by the pair of X and Y coils.

The voltage of the system is typically set at 12 V. This voltage provides a sufficient magnetic field to the workspace, while also preventing the coils from overheating. Due to the system operating on PWM, the magnetic field strength can be further regulated by varying the DC in software. Figure 5c shows the magnetic field generated by each axis as a function of the PWM DC applied to the system at 12 V supply voltage. It is observed that the field increases linearly from 0 mT to approximately 13 mT, 12.5 mT, and 8.3 mT at 100% DC for the x, y, and z coils, respectively. Similarly, Figure 5d shows the magnetic field as a function of duty cycle at 24 V. The field increases from 0 mT to approximately 20 mT, 19.4 mT, and 16 mT at 24V for the *x*, *y* and *z* axis coils, respectively. As a result, it can be seen that the system can generate a significant range of magnetic field strengths depending on the application. These fields are sufficient for actuating a wide variety of magnetic microrobots.

Additionally, the rotating magnetic field of the system was evaluated by measuring a microrobot’s speed as a function of the rotating frequency, as seen in Figure 5e. A 20 *μ*m silica microsphere coated in 250 nm of nickel was used as the testing agent. Experiments were done at a voltage setting of both 12 V and 24 V to compare the microrobots rolling efficiency. For each frequency, three trials were conducted for the 12V experiments, and four trials were conducted for the 24V experiments. The error bars indicate the standard deviation.

Starting at 1 Hz, the rotating magnetic field frequency was increased in 10 Hz increments until reaching a maximum of 100 Hz. At both 12 and 24 V, the microrobot’s speed was approximately the same until reaching 40 Hz. At this frequency, a step-out frequency was observed at 12 V applied to the system. This resulted in a decrease in velocity despite an increase in frequency. The step-out frequency at which magnetic microrobots lose synchronization is affected by several factors, including their magnetic properties, shapes, the strength of the applied field, and the viscosity of the environment [20, 21]. The maximum velocity of the microrobot at 12 V is 160 *μ*m/s with the step-out frequency of 40 Hz. However, at 24 V, due to the increase in power to the system, and thus an increase in magnetic field, the step-out frequency for the same microrobot was found to be much higher at around 70 Hz. As a result, the maximum velocity for the same microrobot at 24 V is 270 *μ*m/s.

### 3.6 | Experimental Validation

#### 3.6.1 | Closed-Loop Path Following of a Rolling Magnetic Microrobot

First, a simple error-minimizing path following algorithm was programmed into the software (see Algorithm 1). The algorithm aims to minimize the distance between a microrobots position $p_{x}$,$p_{y}$ and a target coordinate $\left(x_{i},y_{i}\right)$. This is achieved by calculating an angle that points in the direction of the target coordinate. This angle α is determined using arctan $\left(\frac{\left(y_{i}-p_{y}\right)}{\left(x_{i}-p_{x}\right)}\right)$ and is constantly updated at the same rate as the camera frame rate. If the microrobot comes within a certain threshold distance of the target coordinate, the algorithm will move on to the next coordinate in the trajectory array. This allows the microrobot to follow a path consisting of N nodes or coordinates.

**ALGORITHM 1 |:** Closed-loop Path Following for Rolling Microrobots.

1: **Input:** Trajectory array of n nodes with positions (x,y) 2: **Output:** Magnetic Field Vector $B\leftarrow \left[B_{x},B_{y},B_{z}\right]$ 3: *Initialization:* 4: $trajectory\;\leftarrow \;{\left[\left[x_{0},y_{0}\right],\left[x_{1},y_{1}\right],\ldots ,\left[x_{N},y_{N}\right]\right]}^{⊤}$ 5: i←0 6: f←10 Hz 7: $\gamma \leftarrow 90^{\circ }$ 8: **while** i<N **do** 9: $p\leftarrow \left[p_{x},p_{y}\right]$ 10: **node** ←$\left[x_{i},y_{i}\right]$ 11: $error\;\leftarrow \sqrt{{\left(x_{i}-p_{x}\right)}^{2}+{\left(y_{i}-p_{y}\right)}^{2}}$ 12: $\alpha \leftarrow \arctan\left(\frac{y_{i}-p_{y}}{x_{i}-p_{x}}\right)$ 13: **if** *error*<threshold **then** 14: i←i+1 15: **else** 16: $B_{x}\leftarrow \cos(\gamma )\cos(\alpha )\cos(2\pi ft)+\sin(\alpha )\sin(2\pi ft)$ 17: $B_{y}\leftarrow -\cos(\gamma )\sin(\alpha )\cos(2\pi ft)+\cos(\alpha )\sin(2\pi ft)$ 18: $B_{z}\leftarrow \sin(\gamma )\cos(2\pi ft)$ 19: **Apply B** 20: **end if** 21: **end while**

**FIGURE 6 |:**
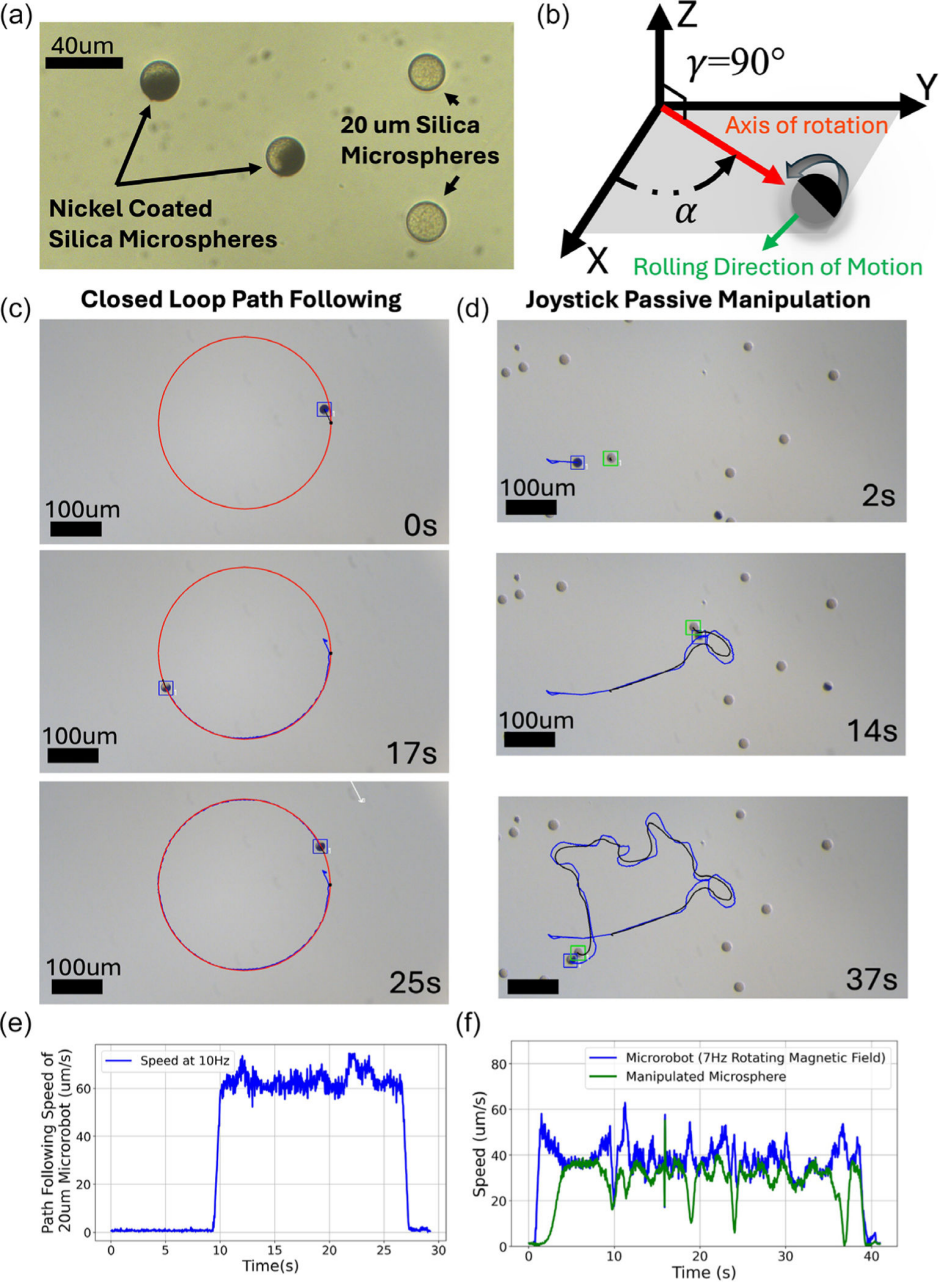
(a) Optical microscopy images of magnetic rolling microrobots composed of 20 *μ*m silica microspheres coated in 250 nm of nickel. (b) Schematic representation demonstrating how the axis of rotation can be adjusted to roll a microrobot forward. 20 *μ*m rolling magnetic microrobot following a circle trajectory using closed-loop control Algorithm 1. (d) Passive manipulation of a 20 *μ*m tracer particle using a 20 *μ*m rolling magnetic microrobot actuated at a rotating magnetic field of 7 Hz and 12 V. (e) Live speed data over time of the rolling microrobot as it follows the red circular path. (f) Live speed data of the microrobot and passive sphere as the microrobot pushes it around the environment.

Figure 6a illustrates 20 *μ*m silica microspheres coated in 250 nm of nickel and uncoated passive silica microspheres. This microrobot is actuated by a rotating magnetic field. Figure 6b illustrates how the axis of the rotating magnetic field can be adjusted in any direction to induce rolling motion of the microrobot. Figure 6c shows the microrobot autonomously following a predefined circular path using Algorithm 1. The red line indicates the desired trajectory, which consists of 100 nodes or target coordinates. The blue line indicates the actual robots trajectory. The goal of the algorithm is to minimize the error between the robots current position and the next node in the trajectory array as described above. The frequency of the rotating magnetic field was set to 10 Hz, and the external power supply was set to 12 V. The microrobot moved at an average velocity of 63.2 *μ*m/s (See Figure 6e). The microrobot took 25 s to complete the path, and the error between the actual and desired path is minimal as seen in Figure 6c and Supplemental Video 1.

#### 3.6.2 | Manual Joystick-Controlled Rolling Microrobotic Passive Microsphere Manipulation

Next, a manual passive particle manipulation experiment was performed using a PS4 dual-shock gaming joystick. Figure 6d shows snapshots of the 20 *μ*m nickel coated microrobot actively pushing a passive 20 *μ*m silica particle while avoiding nearby passive particles. A custom button mapping is used to assign particular actuation variables to the buttons and joysticks of the controller. The right joystick of the controller controls the 0 < *α* < 360° variable, which therefore determines the heading direction of a rolling microrobot. The left joystick controls the field direction and can be used to orient a magnetic microrobot if the microrobot has an inherent self-propulsion mechanism. The left and right triggers of the controller control the negative and positive magnetic field intensity in the z-direction, respectively. The square button on the controller switches *γ* to 180°, resulting in a spinning motion of the microrobot in the clockwise direction. The circle button switches *γ* to 90°, resulting in a spinning motion of the microrobot in the counterclockwise direction. These quick buttons allow for increased control over the behavior of the microrobot when passively manipulating microobjects. In this experiment, the microrobot is tracked using a blue bounding box and blue tracking line. The object to be manipulated is tracked using a green bounding box and a black tracking line. This experiment not only highlights the precision of the system in actively manipulating a passive microparticle using a gaming controller, but also the robustness of the custom embedded tracking and detection algorithm. Over the course of approximately 1 min both the microrobot and manipulated object can be tracked effectively in real-time without any tracking malfunction. The efficiency in tracking enables more sophisticated data-driven micromanipulation algorithms. See Supplemental Video 2 for more details. Figure 6f shows the velocity of both the microrobot and the passive particle over the 40 s experiment.

#### 3.6.3 | Closed-Loop Magnetic and Acoustic Dual Actuation

**FIGURE 7 |:**
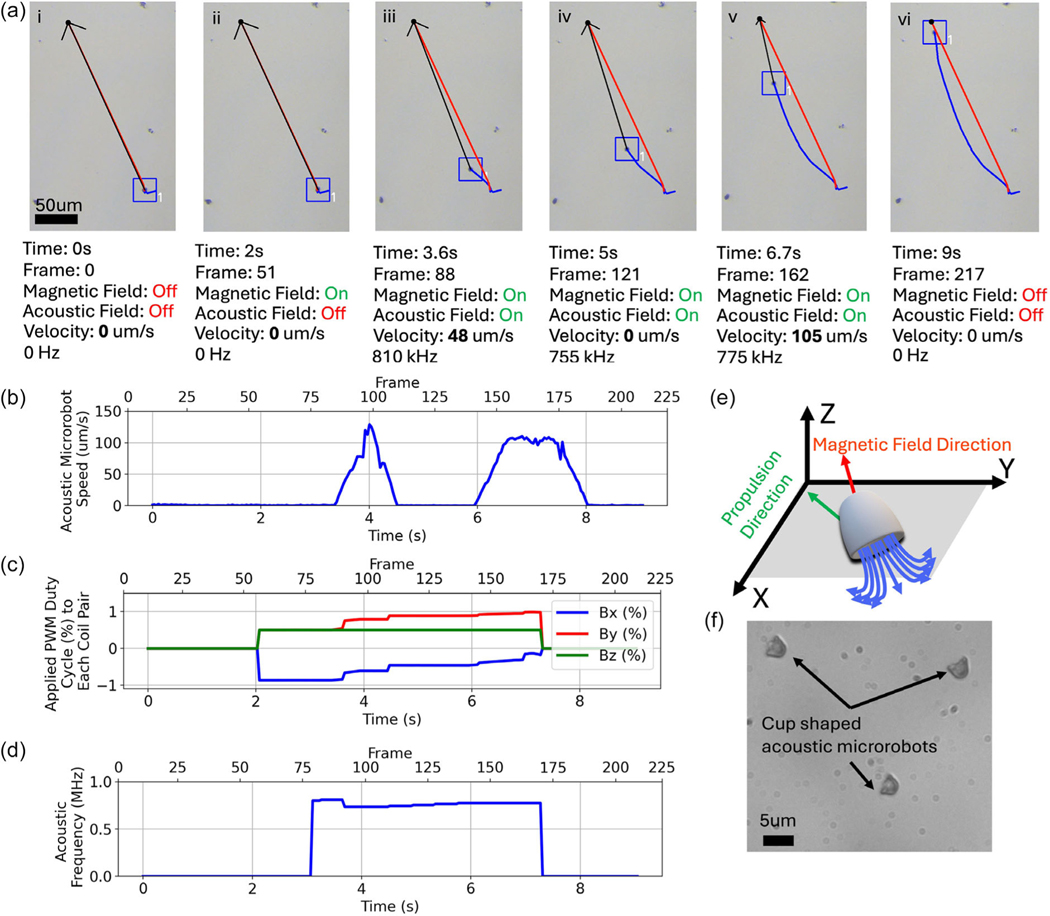
Magnetic and acoustic dual actuation closed-loop targeting experiment. (a) Snapshots of a 3 μm cup-shaped microrobot autonomously being guided toward a target location (black arrow). (b) The resulting linear velocity of the cup-shaped microrobot over time during the experiment. (c) The applied PWM DC is applied to the *x*, *y* and *z*-axis of the coil system at each frame of the experiment. Negative DCs indicate the field was applied in the negative direction. Experiments were conducted at 12 V. (d) Applied an acoustic frequency to the piezoelectric transducer over time during this experiment. (e) Motion schematic of the acoustic microrobot. (f) Optical microscopy images of the cup-shaped microrobots under a 50x objective.

Finally, a closed-loop navigation experiment was performed to validate the simultaneous magnetic and acoustic actuation functionality of the system. A piezoelectric ring transducer measuring 18 mm by 12 mm by 1.2 mm from StemInc (SMR1812T12R412WL) was used. A standard glass microscope slide measuring 75 mm by 25 mm was cut in half, and the transducer was glued to the bottom using UV-curable glue. The transducer was connected to the system using a 2-pin terminal near the top of the coil stand (See Figure 1c). Three *μ*m in size cup-shaped acoustic microrobots from [22] were used as the microrobotic agent. Figure 7f shows optical microscopy images of microrobots. The microrobots were scratched from the surface of a silicon wafer using a pipette tip with water and added to the transducer substrate. The propulsion principle is illustrated in Figure 7d. Figure 5f shows the speed of the cup-shaped robot as the acoustic frequency is swept from 0 MHz to 3 MHz. It is observed that the microrobot’s velocity peaks around 1.2 MHz, 1.8 MHz, and 2 MHz. These peaks and drops in velocity are potentially the result of multiple resonant modes of the microrobot, especially as it moves through the liquid medium. Additionally, the velocity of the microrobot also depends on the microrobot’s orientation. Therefore, its orientation changes as it propels, which can result in unpredictable motion behavior. However, further work is required to fully understand and characterize these frequency-dependent dynamics. Figure 7 and Supplemental Video 3 illustrate the results of the closed-loop autonomous navigation experiment.

A closed-loop orientation control algorithm is used to direct the microrobot toward a target coordinate. It does this by finding the rotation matrix between the microrobot’s current direction of motion and the applied magnetic field direction. Once a target coordinate is defined, it finds the vector from the microrobot’s current position to this target vector. It then applies a magnetic field in the direction of this target vector using this rotation matrix. It does this continuously until the microrobot reaches the target coordinate. The algorithm is described in more detail in [23]. To get the microrobot moving in the first place, an algorithm highlighted in [24] and shown below in Algorithm 2 was implemented into the system to regulate the microrobot’s propulsion velocity.

Figure 7a illustrates snapshots from the experiment with details of the time, frame number, magnetic field status, acoustic field status, velocity of the microrobot, and applied acoustic frequency. In 7ai, at time 0s and frame 0, both the magnetic field and acoustic field are off. Therefore, the velocity of the microrobot is zero. In (ii), the magnetic field is turned on at approximately 2s at frame 51. This begins the orientation algorithm from [23], which orients the microrobot toward a target coordinate indicated by the black arrow. In (iii), 2 begins and finds an acoustic frequency of 810 kHz. The microrobot begins to propel forward, and the magnetic field adjusts itself in real-time to guide the microrobot in the direction of the black target vector. However, in (iv), the acoustic frequency drops to 755 kHz, resulting in the microrobot coming to a stop. The algorithm recognizes that the microrobot has zero velocity despite an applied acoustic frequency. As a result, in (v) the acoustic frequency is autonomously increased to 775 kHz, which resumes the microrobot’s propulsion before finally reaching the target vector head in (vi) and concluding the closed-loop control sequence.

Figure 7b shows a graph of the microrobot’s real-time velocity over time from this closed-loop control sequence. Figure 7b depicts a graph of the PWM DC percentage applied to the x, y, and z pair of coils over time based on the orientation algorithm. This is proportional to the magnetic field applied in each direction. A negative percentage indicates a field was applied in the negative direction. During the experiment, the PWM DC in the z-direction is set to 50% which allows the microrobot to orient itself slightly upwards. Finally, Figure 7c depicts a graph of the acoustic frequency applied to the piezoelectric transducer over time. These frequencies are adjusted based on the microrobot’s velocity from 2.

**ALGORITHM 2 |:** Acoustic Microrobot Automated Control Algorithm.

1: **Input**: Microrobot’s Velocity Magnitude $v_{mag}$ 2: **Output**: Acoustic Wave Generator Frequency $f_{current}$ 3: **Initialization**: 4: Define $f_{\min}$ 5: Define $f_{\max}$ 6: $f_{current}\leftarrow f_{\min}$ 7: Define $v_{\min}$ 8: Define $v_{\max}$ 9: increment←0.1 MHz 10: $f_{optimal}\leftarrow \;\text{None}\;$ 11: n←0 12: **while** True **do** 13: **if** $v_{\mathrm{mag}}$ < $v_{\text{min}}$ **then** 14: **if** $f_{current}<f_{\max}$ **then** 15: **if** nmod10=0 **then** 16: $f_{current}\leftarrow f_{\text{current}\;}+\;increment$ 17: **end if** 18: **else if** $f_{current}\geq f_{\max}$ **then** 19: increment←increment/2 20: $f_{current}\leftarrow f_{\min}$ 21: **end if** 22: **else if** $v_{\min}<v_{mag}<v_{\max}$ **then** 23: $f_{optimal}\leftarrow f_{current}$ 24: increment←increment/10 25: **else if** $v_{mag}>v_{\text{max}\;}$ **then** 26: **if** nmod20=0 **and** $f_{current}>f_{\min}$ **then** 27: $f_{current}\leftarrow f_{current}-0.075$ MHz 28: **end if** 29: **end if** 30: n←n+1 31: **end while**

## 4 | Conclusion

This paper presents a versatile and portable platform for data-driven acoustic and magnetic microrobotic experimentation. All system-wide mechanical, electrical, and software components involved in constructing the instrument were described. Then we outline the magnetic field generation method.

Next, we provide magnetic field measurements at various system settings at the center of the workspace. To validate the system’s experimental versatility, we experiment with several representative microrobotic operations commonly found in the literature. These include autonomous path following of a rolling microrobot and joystick-controlled passive particle manipulation. We also demonstrate vision-based acoustic and magnetic microrobot navigation enabled by the simultaneous generation and control of both fields.

In the short term, the current functionality of the MicroRoboScope architecture can enable more sophisticated machine learning control policies due to the real-time tracking and output action data recording pipelines. Additionally, more sophisticated, learning-based tracking and detection algorithms such as YOLO [25] can be implemented for unique microrobotic tracking or single cell tracking, enabling more advanced single cell manipulation methods. The same hardware–software control architecture has already enabled a learning based microrobotic control strategy outlined in [26], a hybrid acoustic magnetic microswimmer targeting algorithm outlined above and in [24], and a novel micro-object and single cell pushing algorithm outlined in [27]. Each of these control strategies and applications can be easily implemented on the MicroRoboScope. Future work should also include integrating biological incubation features such as $CO_{2}$, humidity, and temperature control features. This would allow for long-time scale biological and microrobotic hybrid experiments for tissue engineering applications.

In conclusion, this platform offers a powerful and efficient solution for microrobotic experimentation by integrating key microrobotic functionality, thereby lowering the barrier to entry for microrobotic beginners. Additionally, a user-friendly control system of this size and functionality could be used in a microrobotic lab-based graduate course similar to [28]. Overall, the work represents a significant step toward transitioning microrobotic systems from specialized laboratory setups to broader research, commercial, and educational environments.

## Supplementary Material

supplementary information

video 1

video 2

video 3

video 4

Supporting Information

Additional supporting information can be found online in the Supporting Information section.

## Figures and Tables

**TABLE 1 |:** Hardware and integration comparison between ModMag and MicroRoboScope.

Feature	ModMag	MicroRoboScope
Primary function	Touchscreen device for generating fields to external magnetic actuation systems	Full microscopy, actuation, tracking, and data recording integration
Primary compute	Raspberry Pi Model 3B+	Jetson AGX Orin + Arduino Mega 2560
Magnetic drivers	Six BTS7960B H-bridge PWM drivers	Six BTS7960B H-bridge PWM drivers
Power delivery	12 V battery pack (peak 3 A)	External PSU 0–27 V, 0–20 A
Acoustic actuation	AD9850 + piezoelectric transducer	AD9850 + piezoelectric transducer
Coil configuration	external coil setups	embedded coil setup
Magnetic actuation	Uniform + gradient + rotating fields.	Uniform + gradient + rotating fields.
Microscopy	None	Embedded XYZ stage + camera + objective + illumination
Software	Python: Tkinter, gpiozero	Python: PyQT, OpenCV, pySerialTransfer, pygame
Feedback	None	Camera + hall effect sensors
Footprint	100 × 150 × 250 mm	240 × 280 × 350 mm

## Data Availability

All data is contained in this manuscript. The open-source control software can be found on GitHub here: https://github.com/MaxSokolich/Magnetoacoustic-Microrobotic-Manipulation-System.
