# Supplementary material for: MicroRoboScope: A Portable and Integrated Mechatronic Platform for Magnetic and Acoustic Microrobotic Experimentation
Source: Adv Robot Res. Author manuscript; Available in PMC 2026 Jul 18. (PMC13378525; doi:10.1002/adrr.202500195)
Supplement: supplementary information [file NIHMS2178189-supplement-supplementary_information.pdf]

Supplementary Information

July 26, 2025

1 Video Descriptions

- **Video 1:** 20 µm silica microsphere coated in 250 nm of Ni following a predefined circle trajectory using closed loop control algorithm. Actuating at a rotating magnetic field frequency of 10 Hz and 12V.
- **Video 2:** Manual micro-particle manipulation of a 20 um microsphere using a the same microrobot described in video 1. Controlling the heading angle α using the joystick of a PS4 gaming controller at a rotating magnetic field frequency of 7 Hz and 12V.
- **Video 3:** Acoustic 3 um cup shaped microrobot autonomously targeting a predefined approach coordinate by adjusting the magnetic field and acoustic frequency in real time.

2 Arduino Code

The following Arduino code is used to receive information from the Python GUI and generate the appropriate PWM signals to generate arbitrary magnetic fields and also to interface with the AD9850 DDS module. The code also interfaces with the ADS1115 ADC and sends magnetic field sensor data back to the Python GUI.

```
1
2  #include <AD9850.h>
3  #include <Wire.h>
4  #include "Adafruit_ADS1X15.h"
5  #include "SerialTransfer.h"
6
7
8  Adafruit_ADS1115 ads;  // Create ADS1115 object
9  unsigned long lastReadTime = 0;  // Store the time of the last read
10 unsigned long readInterval = 1000; // 1 s interval
11
12 SerialTransfer myTransfer;
13
14
```

```
float action[9]; //an array to store incoming data from python


//Store data from arduino to send to python
struct send_class {
  float Bx_sensor;
  float By_sensor;
  float Bz_sensor;

} send_data;



#define PI 3.14159265358979323846264338327795

//actions
float Bx;
float By;
float Bz;
float alpha;
float gamma;
float rolling_frequency;
float psi;
float gradient_status;
float acoustic_frequency;




int phase = 0;



//other field vals
float Bx_roll;
float By_roll;
float Bz_roll;

float Bx_uniform;
float By_uniform;
float Bz_uniform;

float BxPer;
float ByPer;
float BzPer;
float c;
float magnitude;
```

```
62
63
64 //other constants
65 float tim;
66 float t;
67 float omega;
68
69 float Bx_final;
70 float By_final;
71 float Bz_final;
72
73 // calibration params for hall effect sensor values
74 float zeroFieldVoltagex = 2.476;
75 float sensitivityx = 0.0069;
76
77 float zeroFieldVoltagey = 2.495;
78 float sensitivityy = 0.0059;
79
80 float zeroFieldVoltagez = 2.525;
81 float sensitivityz = 0.002;
82
83 int16_t hall1 = 0;
84 int16_t hall2 = 0;
85 int16_t hall3 = 0;
86
87
88
89 //Coil 1 : +Y Brown
90 const int Coil1_PWMR = 2;
91 const int Coil1_PWML = 3;
92 const int Coil1_ENR = 26;
93 const int Coil1_ENL = 27;
94 //Coil 2 : +X Purple
95 const int Coil2_PWMR = 44;
96 const int Coil2_PWML = 5;
97 const int Coil2_ENR = 24;
98 const int Coil2_ENL = 25;
99
100 //Coil 3 : -Y Green
101 const int Coil3_PWMR = 6;
102 const int Coil3_PWML = 7;
103 const int Coil3_ENR = 22;
104 const int Coil3_ENL = 23;
105
106 //Coil 4: -X Blue
107 const int Coil4_PWMR = 8;
108 const int Coil4_PWML = 9;
```

```
const int Coil4_ENR = 32;
const int Coil4_ENL = 33;

//Coil 5 : +Z  Yellow
const int Coil5_PWMR = 10;
const int Coil5_PWML = 11;
const int Coil5_ENR = 30;
const int Coil5_ENL = 31;

//Coil 6 : -Z Orange
const int Coil6_PWMR = 12;
const int Coil6_PWML = 46;
const int Coil6_ENR = 28;
const int Coil6_ENL = 29;


//AD9850 Acoustic Module
const int W_CLK_PIN = 34;
const int FQ_UD_PIN = 36;
const int DATA_PIN = 38;
const int RESET_PIN = 40;



void setup()
{


  cli();
  TCCR1B = (TCCR1B & 0b11111000) | 0x01; //31.37255 [kHz] pin 12,11
  TCCR2B = (TCCR2B & 0b11111000) | 0x01; //31.37255 [kHz] pin 10,9
  TCCR3B = (TCCR3B & 0b11111000) | 0x01; //31.37255 [kHz] pin 5,3,2
  TCCR4B = (TCCR4B & 0b11111000) | 0x01; //31.37255 [kHz] pin 8,7,6
  TCCR5B = (TCCR5B & 0b11111000) | 0x01; //31.37255 [kHz] pin
     44,45,46
  sei();

  Serial.begin(115200);
  myTransfer.begin(Serial);

    //start acoustic module
  DDS.begin(W_CLK_PIN, FQ_UD_PIN, DATA_PIN, RESET_PIN);
  DDS.calibrate(124999500);

  ads.begin();


```

```
155
156     //Coil1 Ouptut
157   pinMode(Coil1_PWMR, OUTPUT);
158   pinMode(Coil1_PWML, OUTPUT);
159   pinMode(Coil1_ENR, OUTPUT);
160   pinMode(Coil1_ENL, OUTPUT);
161
162
163
164   //Coil2 Output
165   pinMode(Coil2_PWMR, OUTPUT);
166   pinMode(Coil2_PWML, OUTPUT);
167   pinMode(Coil2_ENR, OUTPUT);
168   pinMode(Coil2_ENL, OUTPUT);
169
170
171
172   //Coil3 Output
173   pinMode(Coil3_PWMR, OUTPUT);
174   pinMode(Coil3_PWML, OUTPUT);
175   pinMode(Coil3_ENR, OUTPUT);
176   pinMode(Coil3_ENL, OUTPUT);
177
178
179   //Coil4 Output
180   pinMode(Coil4_PWMR, OUTPUT);
181   pinMode(Coil4_PWML, OUTPUT);
182   pinMode(Coil4_ENR, OUTPUT);
183   pinMode(Coil4_ENL, OUTPUT);
184
185
186   //Coil5 Output
187   pinMode(Coil5_PWMR, OUTPUT);
188   pinMode(Coil5_PWML, OUTPUT);
189   pinMode(Coil5_ENR, OUTPUT);
190   pinMode(Coil5_ENL, OUTPUT);
191
192
193   //Coil6 Output
194   pinMode(Coil6_PWMR, OUTPUT);
195   pinMode(Coil6_PWML, OUTPUT);
196   pinMode(Coil6_ENR, OUTPUT);
197   pinMode(Coil6_ENL, OUTPUT);
198
199
200 }
201
```

```
void set1(float DC1){
  digitalWrite(Coil1_ENR,HIGH);
  digitalWrite(Coil1_ENL,HIGH);

  if (DC1 > 0){
    analogWrite(Coil1_PWMR,abs(DC1)*255);
    analogWrite(Coil1_PWML,0);
  }
  else if (DC1 < 0){
    analogWrite(Coil1_PWMR,0);
    analogWrite(Coil1_PWML,abs(DC1)*255);
  }
  else {
    analogWrite(Coil1_PWMR,0);
    analogWrite(Coil1_PWML,0);
  }
}


void set2(float DC2){
  digitalWrite(Coil2_ENR,HIGH);
  digitalWrite(Coil2_ENL,HIGH);

  if (DC2 > 0){
    analogWrite(Coil2_PWMR,abs(DC2)*255);
    analogWrite(Coil2_PWML,0);
  }
  else if (DC2 < 0){
    analogWrite(Coil2_PWMR,0);
    analogWrite(Coil2_PWML,abs(DC2)*255);
  }
  else {
    analogWrite(Coil2_PWMR,0);
    analogWrite(Coil2_PWML,0);
  }
}


void set3(float DC3){
  digitalWrite(Coil3_ENR,HIGH);
  digitalWrite(Coil3_ENL,HIGH);

  if (DC3 > 0){
    analogWrite(Coil3_PWMR,abs(DC3)*255);
    analogWrite(Coil3_PWML,0);
  }
  else if (DC3 < 0){
```

```
249     analogWrite(Coil3_PWMR,0);
250     analogWrite(Coil3_PWML,abs(DC3)*255);
251   }
252   else {
253     analogWrite(Coil3_PWMR,0);
254     analogWrite(Coil3_PWML,0);
255   }
256 }
257
258
259 void set4(float DC4){
260   digitalWrite(Coil4_ENR,HIGH);
261   digitalWrite(Coil4_ENL,HIGH);
262
263   if (DC4 > 0){
264     analogWrite(Coil4_PWMR,abs(DC4)*255);
265     analogWrite(Coil4_PWML,0);
266   }
267   else if (DC4 < 0){
268     analogWrite(Coil4_PWMR,0);
269     analogWrite(Coil4_PWML,abs(DC4)*255);
270   }
271   else {
272     analogWrite(Coil4_PWMR,0);
273     analogWrite(Coil4_PWML,0);
274   }
275 }
276
277
278 void set5(float DC5){
279   digitalWrite(Coil5_ENR,HIGH);
280   digitalWrite(Coil5_ENL,HIGH);
281
282   if (DC5 > 0){
283     analogWrite(Coil5_PWMR,abs(DC5)*255);
284     analogWrite(Coil5_PWML,0);
285   }
286   else if (DC5 < 0){
287     analogWrite(Coil5_PWMR,0);
288     analogWrite(Coil5_PWML,abs(DC5)*255);
289   }
290   else {
291     analogWrite(Coil5_PWMR,0);
292     analogWrite(Coil5_PWML,0);
293   }
294 }
295
```

```
296
297 void set6(float DC6){
298   digitalWrite(Coil6_ENR,HIGH);
299   digitalWrite(Coil6_ENL,HIGH);
300
301   if (DC6 > 0){
302     analogWrite(Coil6_PWMR,abs(DC6)*255);
303     analogWrite(Coil6_PWML,0);
304   }
305   else if (DC6 < 0){
306     analogWrite(Coil6_PWMR,0);
307     analogWrite(Coil6_PWML,abs(DC6)*255);
308   }
309   else {
310     analogWrite(Coil6_PWMR,0);
311     analogWrite(Coil6_PWML,0);
312   }
313 }
314
315
316 void loop()
317
318
319 {
320         unsigned long currentTime = millis();
321         if (currentTime - lastReadTime >= readInterval) {
322             hall1 = ads.readADC_SingleEnded(1);  // Sensor 2 on A1
                    ---> purple wire goes to Pin 9 on the connector
                    terminal which is connected to the Z hall sensor
323             hall2 = ads.readADC_SingleEnded(2);  // Sensor 3 on A2
                    ---> blue wire goes to Pin 7 on the connector
                    terminal which is connected to the Y hall sensor
324             hall3 = ads.readADC_SingleEnded(3);  // Sensor 3 on A3
                    ---> blue wire goes to Pin 5 on the connector
                    terminal which is connected to the X hall sensor
325
326             lastReadTime = currentTime;  // Update the last read
                    time
327   }
328
329
330         // Convert raw reading to voltage if needed:
331         float voltage1 = hall1 * 0.1875 / 1000;  // Default gain =
                6 .144V, LSB = 0.1875mV
332         float voltage2 = hall2 * 0.1875 / 1000;
333         float voltage3 = hall3 * 0.1875 / 1000;
334
```

```
335         float magneticField_Gx = (voltage3 - zeroFieldVoltagex) /
               sensitivityx;
336         float magneticField_Gy = (voltage2 - zeroFieldVoltagey) /
               sensitivityy;
337         float magneticField_Gz = (voltage1 - zeroFieldVoltagez) /
               sensitivityz;
338
339         float magneticField_mTx = magneticField_Gx *.1;
340         float magneticField_mTy = magneticField_Gy *.1;
341         float magneticField_mTz = magneticField_Gz *.1;
342
343
344
345         send_data.Bx_sensor = magneticField_mTx;
346         send_data.By_sensor = magneticField_mTy;
347         send_data.Bz_sensor = magneticField_mTz;
348
349
350
351     if  (myTransfer.available()){
352
353               uint16_t recSize = 0;
354               recSize = myTransfer.rxObj(action,recSize);
355
356
357               uint16_t sendSize = 0;
358               sendSize = myTransfer.txObj(send_data, sendSize);
359               myTransfer.sendData(sendSize);
360
361
362     }
363
364
365    //LOGIC
366    Bx_uniform = action[0];  \\ -1 to 1
367    By_uniform = action[1];  \\ -1 to 1
368    Bz_uniform = action[2];  \\ -1 to 1
369    alpha = action[3];       \\ 0 to 2pi
370    gamma = action[4];       \\ 0 to pi
371    rolling_frequency = action[5];  \\ 0 to 250 Hz
372    psi = action[6];                \\ 0 to pi/2
373    acoustic_frequency = action[7]; \\ 0 to Sin40MHz
374    gradient_status = action[8];    \\ 0  or 1. 1
375
376
377
378
```

```
379
380     if (acoustic_frequency != 0){
381        DDS.setfreq(acoustic_frequency, phase);
382     }
383     else{
384        DDS.down();
385     }
386
387
388     omega = 2*PI*rolling_frequency;
389
390
391
392     t = micros() / 1e6;
393
394     //tim = micros() % 7812500;
395     //t = tim / 7812500;
396
397     if (omega == 0){
398         Bx_roll = 0;
399         By_roll = 0;
400         Bz_roll = 0;
401        }
402     else {
403        //working equations
404        Bx_roll = - (cos(alpha) * cos(gamma)  * cos(omega*t))     +
                  (sin(alpha) * sin(omega*t)) ;
405        By_roll = - (sin(alpha) * cos(gamma)  * cos(omega*t))     -
                  (cos(alpha) * sin(omega*t));
406        Bz_roll = sin(gamma) * cos(omega*t);
407
408         // condition for perpendicular field (psi cannot be 90)
409         // condition for perpendicular field (psi cannot be 90)
410        if (psi < PI/2){
411            c = 1/tan(psi);
412            BxPer = c* cos(alpha) * sin(gamma);
413            ByPer = tan(alpha) * BxPer;
414            BzPer = BxPer * (1/cos(alpha)) * (1/tan(gamma));
415            }
416        else{
417            c = 0;
418            BxPer = 0;
419            ByPer = 0;
420            BzPer = 0;
421        }
422
423         // superimpose the rolling field with the perpendicular field
```

```
424         Bx_roll = (Bx_roll + BxPer) / (1+c);
425         By_roll = (By_roll + ByPer) / (1+c);
426         Bz_roll = (Bz_roll + BzPer) / (1+c);
427
428         }
429     //need to add unform field with rotating field and normalize
430     //cc = sqrt(Bx_roll*Bx_roll + By_roll*By_roll + Bz_roll*Bz_roll)
431     Bx = (Bx_uniform + Bx_roll); /// (1+cc);
432     By = (By_uniform + By_roll); /// (1+cc);
433     Bz = (Bz_uniform + Bz_roll); //// (1+cc);
434
435
436     // condition to prevent divide by zero error when total Bx, By,
           Bz are off aka zeroed
437     if (Bx == 0 and By == 0 and Bz ==0){
438         Bx_final = 0;
439         By_final = 0;
440         Bz_final = 0;
441     }
442     // otherwise I need to normalize the superpoistion of the
           rotating field with the uniform field
443     else{
444         magnitude = max(sqrt(Bx_uniform*Bx_uniform + By_uniform*
               By_uniform + Bz_uniform*Bz_uniform),
445                         sqrt(Bx_roll*Bx_roll + By_roll*By_roll +
                               Bz_roll*Bz_roll));
446
447
448         Bx_final = magnitude * (Bx / sqrt(Bx*Bx + By*By + Bz*Bz));
449         By_final = magnitude * (By / sqrt(Bx*Bx + By*By + Bz*Bz));
450         Bz_final = magnitude * (Bz / sqrt(Bx*Bx + By*By + Bz*Bz));
451
452
453     }
454
455
456   // if gradient status = 1: output the the corresponding gradient
         field
457     if (gradient_status != 0){
458         //y gradient
459         if (By_final < 0){
460           set1(By_final);
461
462         }
463         else if (By_final > 0){
464           set3(By_final);
465         }
```

```
466         else{ //if By==0
467           set1(0);
468           set3(0);
469         }
470 
471         //x gradient
472         if (Bx_final > 0){
473           set2(Bx_final);
474         }
475         else if (Bx_final < 0){
476           set4(Bx_final);
477         }
478         else{ //if Bx==0
479           set2(0);
480           set4(0);
481         }
482 
483         //z gradient
484         if (Bz_final > 0){
485           set5(Bz_final);
486         }
487         else if (Bz_final < 0){
488           set6(Bz_final);
489         }
490         else{ //if Bz==0
491           set5(0);
492           set6(0);
493         }
494     }
495 
496     // if gradient status = 0: output the correspending uniform field
         by setting opposite facing coils with opposite signed signals
        .
497 
498     else {
499         set1(By_final);
500         set2(Bx_final);
501         set3(-By_final);
502         set4(-Bx_final);
503         set5(Bz_final);
504         set6(-Bz_final);
505     }
506 
507 
508 
509 
510 
```

```
511
512
513         }
```

Listing 1: Arduino code for PWM signal with variable duty cycle.

3 Electrical Schematic

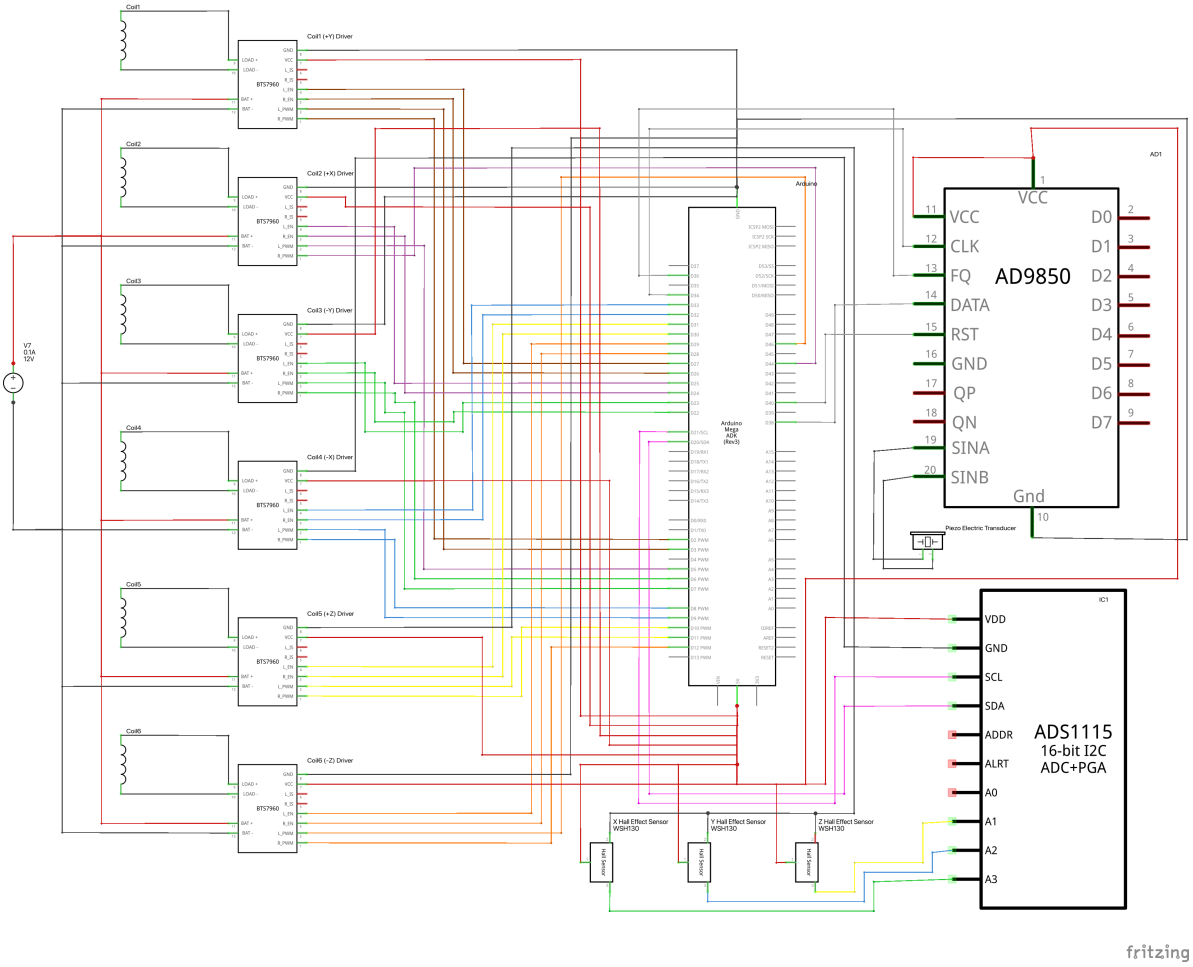

Figure 1: Arduino Electrical Schematic of the System.

4 Oscilloscope AD9850 Waveform output at 1 MHz

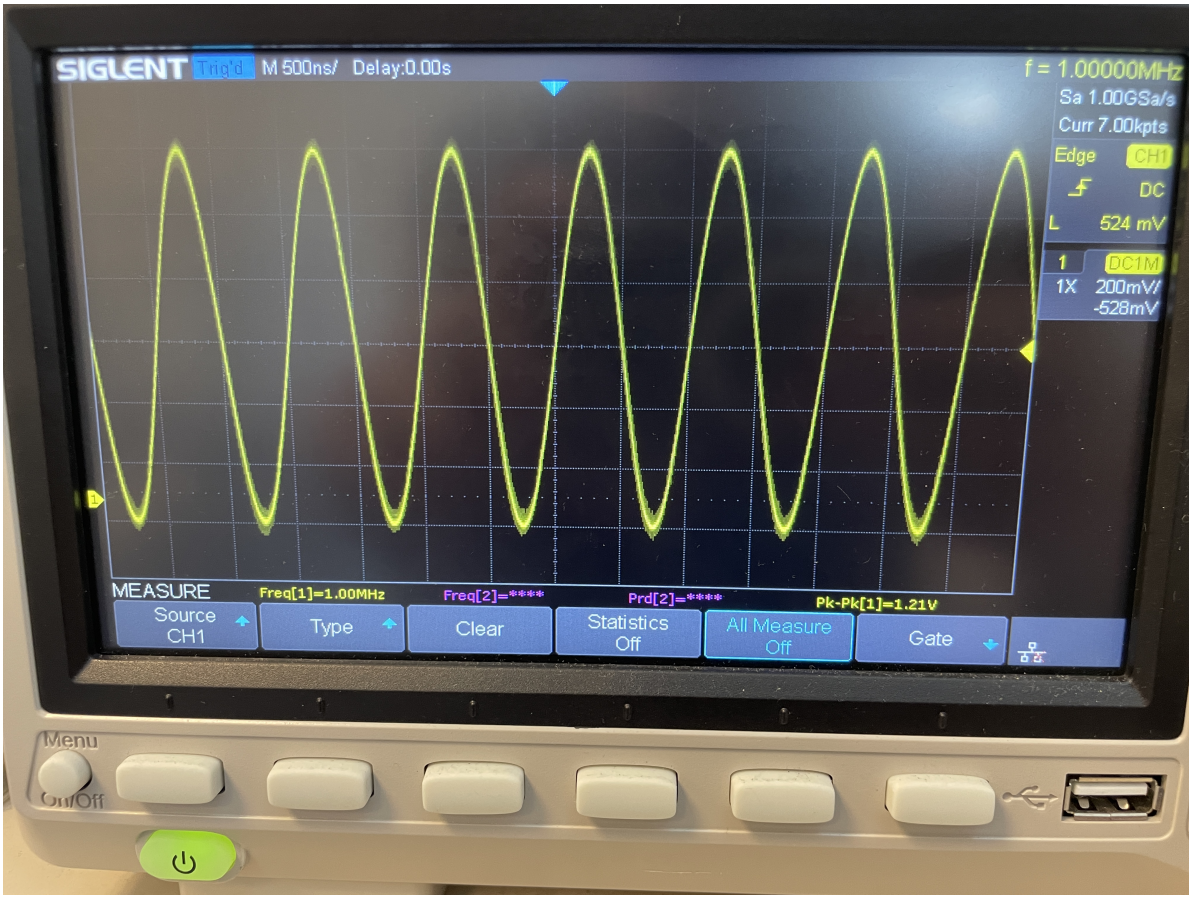

Figure 2: AD9850 1 MHz sine wave output with a peak to peak voltage of 1.21V
